# Perspective on the Application of Erythrocyte Liposome-Based Drug Delivery for Infectious Diseases

**DOI:** 10.3390/membranes12121226

**Published:** 2022-12-03

**Authors:** Hannah Krivić, Sebastian Himbert, Maikel C. Rheinstädter

**Affiliations:** 1 Department of Physics and Astronomy, McMaster University, 1280 Main Street West, Hamilton, ON L8S 4M1, Canada; 2 Origins Institute, McMaster University, Hamilton, ON L8S 4M1, Canada

**Keywords:** nanoparticles, red blood cell liposomes, erythrocyte liposomes, targeted drug delivery, infectious diseases, antibiotics, immunization, neurodegenerative diseases

## Abstract

Nanoparticles are explored as drug carriers with the promise for the treatment of diseases to increase the efficacy and also reduce side effects sometimes seen with conventional drugs. To accomplish this goal, drugs are encapsulated in or conjugated to the nanocarriers and selectively delivered to their targets. Potential applications include immunization, the delivery of anti-cancer drugs to tumours, antibiotics to infections, targeting resistant bacteria, and delivery of therapeutic agents to the brain. Despite this great promise and potential, drug delivery systems have yet to be established, mainly due to their limitations in physical instability and rapid clearance by the host’s immune response. Recent interest has been taken in using red blood cells (RBC) as drug carriers due to their naturally long circulation time, flexible structure, and direct access to many target sites. This includes coating of nanoparticles with the membrane of red blood cells, and the fabrication and manipulation of liposomes made of the red blood cells’ cytoplasmic membrane. The properties of these erythrocyte liposomes, such as charge and elastic properties, can be tuned through the incorporation of synthetic lipids to optimize physical properties and the loading efficiency and retention of different drugs. Specificity can be established through the anchorage of antigens and antibodies in the liposomal membrane to achieve targeted delivery. Although still at an early stage, this erythrocyte-based platform shows first promising results in vitro and in animal studies. However, their full potential in terms of increased efficacy and side effect minimization still needs to be explored in vivo.

## 1. The Status Quo: Current Day Use of Nano Carriers in Drug Delivery

Nanocarriers have been introduced as a solution to the barriers of random distribution, low bioavailability, toxic side effects, and rapid degradation of current drug formulations. The goal of drug delivery technologies is to improve patient health by enhancing the delivery of a therapeutic to its target site, minimizing off-target accumulation, and facilitating patient compliance [1]. A few decades ago, small-molecule drugs were the primary class of therapeutics. Over time, new generations of therapeutics, including proteins and peptides, monoclonal antibodies, nucleic acids, and live cells have provided new therapeutic functions. For all drugs, the goal of delivery is to maximize therapeutic efficacy by transporting and releasing the drug (passively or actively) to the target site in the body and by minimizing off-target accumulation of the drug. These systems include hydrogels, polymeric implants, microparticles, and nanoparticles, which allow for particle-surface modifications to enhance drug half-life and the targeting of particular tissues through specific interactions with the microenvironment.

Nanoparticle drug delivery systems have been used in the clinical setting since the early 1990s. More than 30 nanoparticles platforms have been approved by the Food and Drug Administration (FDA) or European Medicines Agency (EMA) and more than 120 systems are in or have entered clinical trials [2,3]. Oral, local, topical, and intravenous administration have been approved by the FDA for the delivery of nanoparticles/microparticles. Intravenous administration of nanoparticles is the most promising as nanoparticles delivered systemically have direct access to nearly all parts of the body and have the most potential to influence clinical care [4]. However, systemically delivered nanoparticles also face exceedingly difficult challenges with regards to both the delivery aspect and the regulatory aspect and approval challenges. Nanoparticles possess advantages over many intravenously administered pharmaceuticals and biologics. Many of the currently approved and clinically investigated nanoparticles are polyethylene glycol (PEG) terminated or PEGylated which limits interactions with, and rapid clearance by, immune cells. In doing so, nanoparticles can remain in circulation for longer periods of time and increase their chances of reaching and entering target sites.

While nanomedicine holds potential to improve anticancer therapy, patients often only benefit from nanomedicines in clinical practice because of reduced or altered side effects. Despite the approval of an increasing number of nanomedicinal anticancer drugs, the success rate of clinical translation remains relatively low [5]. There is a striking imbalance between the ever-increasing number of preclinical studies reporting the development of ever more complex nanomedicines on the one hand, and the relatively small number of nanomedicine products approved for clinical use on the other. Of the nanomedicines that are approved, few are recommended as first- line treatment options, and many show improvements in only a small subset of patients. This is due, in part, to the underexplored heterogeneity both in the biological underpinnings of diseases and among patients, which alters efficacy because the growth, structure, and physiology of diseased tissue alter nanoparticle distribution and functionality [6].

Applications of synthetic drug delivery systems can be limited due to inefficiency, cytotoxicity and/or immunogenicity [7]. When carried by a delivery system, the clearance and tissue distribution profile of a therapeutic are mainly governed by the characteristics of the vehicle rather than the physicochemical properties of the drug molecule. In this respect, biomimetic drug delivery system mimic the unique structures, functions, and biosynthetic pathways of biological systems (whole cells, structures or composition of cell membrane, and the natural budding processes of exosomes) [8]. Advantages are their high biocompatibility, low immunogenicity, long systematic circulation, and lesion targeting. Systems include cell membrane-camouflaged nanoparticles, extracellular vesicles, lipoprotein-coated nanoparticles, and virus-like nanoparticles. The aim of this perspective is to discuss endogenous, red blood cell based carriers and their potential advantages over existing synthetic and biomimetic platforms. In theory they should have the potential to overcome some of their limitations in terms of biocompatibility, delivery efficiency and heterogeneity, which has yet to be proven in clinical trials.

## 2. The Problem: Limitations of Synthetic Nanocarriers

Nanocarriers show promise as molecules can be conjugated to their surface or encapsulated inside to achieve improved drug stability, targeted delivery, and uniform distribution in the target organ to reduce side effects [9,10,11]. Moreover, ligands or proteins can be conjugated to the surface to achieve targeted delivery thereby reducing the random distribution of the therapeutic molecule throughout the body. If the desired drug is retained in the nanocarrier efficiently, sustained release can be established; the drug can be retained in the nanocarrier for long periods in circulation, allowing for greater doses to be administered to the target. The two main nanocarriers widely accepted in clinical settings are nanoparticles and liposomes.

Nanoparticles can be further classified according to their physical and chemical properties, and fall mainly under the two categories: inorganic or organic nanoparticles. They can be optimized by modifying the size, shape, charge, and hydrophobicity to achieve the most efficient delivery system [3]. Nanoparticle-incorporated Natural killer T cell ligands have shown potential as an immunoadjuvant in the formulation of vaccines [12]. Their versatile properties allow for a wide range of applications such as creating contrast in ultrasound and MRI imaging, cancer treatment, and triggered release delivery systems. Despite their potential, one of the major concerns regarding nanoparticles is related to their toxicity. Inorganic nanoparticles, in particular, have been shown to accumulate in areas such as the spleen, liver, and kidney where they may initiate cell lysis and inflammation, DNA damage, and oxidative stress [13].

**Figure 1:**
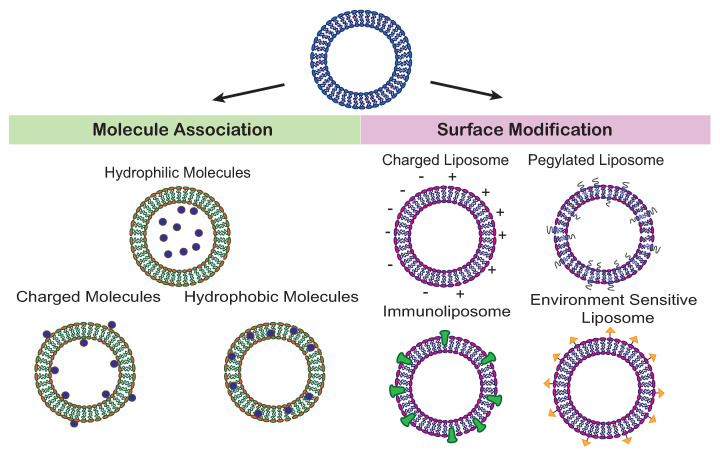
(**Left**) Liposomes can be loaded with molecules of different properties. Hydrophilic molecules are preferably located in the aqueous core, hydrophobic molecules localize within the membrane, and charged molecules may associate with the membrane surface. (**Right**) Surface properties can be tuned by charged lipids, lipids with a bound polymer, e.g., polyethylene glycol (PEG), lipids functionalized with ligands (yellow), and incorporated proteins (green).

Liposomes are composed of phospholipids that form one or more bilayers that can accommodate hydrophobic or lipophilic drugs, and an internal aqueous phase where hydrophilic drugs can be encapsulated. The different systems are sketched in Figure 1. They can be designed using lipids abundant in cellular membranes to improve biocompatibility and to enhance the interaction between liposome and target cell. In this sense, the ability to mimic biological membranes makes liposomes superior to nanoparticles as they do not have to be extensively modified to achieve biocompatability and have minimal toxicity. Phospholipids with varying degrees of charge can further be used to enhance retention of charged molecules by making them attractive to the lipid membrane. By using phospholipids with different properties, the size, composition, and fluidity of the liposome can be modified to obtain the most optimal drug carrier. Through incorporation of a tetrapeptide, Tuftsin-bearing liposomes demonstrated greater immunogenicity by increasing the T cell proliferation and antibody secretion [14]. Immunoliposomes can be produced through the incorporation of antibodies to the liposome’s exterior to achieve targeted delivery. Additionally, incorporation of particular ligands can produce liposomes responsive to certain environmental stimuli, allowing for drug release in specific environmental conditions throughout the body.

Despite the advantages that liposomes offer in drug-delivery, liposomes are still highly susceptible to rapid clearance through the reticuloendothelial system (RES), and their accumulation in the liver and spleen that limits the dose that reaches the target site [7]. Researchers have found that coating the liposome in polyethylene glycol (PEG) produces long-circulating drug-carriers with increased stability. Additional known limitations of liposomal drug delivery are chemical instability during storage, physical instability under physiological conditions that potentially lead to drug leakage and low encapsulation efficiencies [15].

## 3. Erythrocyte-Based Carriers Have Advantages (If They Work)

Red blood cells (RBCs) should have a high potential as drug carriers, as they have high biocompatibility and can prolong the life of drugs in circulation for weeks [16]. The challenge with modifying RBCs is that they may be taken up by macrophages in the spleen and liver, where they undergo lysosomal degradation. Drugs and nanocarriers can be coupled to the RBC surface to allow for hitch-hiking to target sites accessible by the RBCs and further redistribution from blood to plasma. The conjugation of drugs to the RBC surface shows promise, as it reduces damage, retains high RBC biocompatibility, and enhances pharmacokinetics. Small drugs that can diffuse through the membrane tend to be more active when they are coupled to the surface, and RBCs were found to effectively deliver drugs to intravascular targets and targets in the reticuloendothelial system. However, delivery of RBCs to other tissues is limited due to inaccessibility [17,18]. For instance, thrombomodulin (TM) conjugated to RBCs can effectively inhibit clot formation with improved pharmacodynamics and bioavailability compared to free TM [19]. Many therapeutic proteins are limited by the humoral immune response and are thus not used in clinical settings. However, by binding to erythrocytes, the immunological tolerance to *Escherichia coli* L-asparaginase-II (ASNase) was increased [20], indicating that enzyme conjugation to erythrocytes can enhance pharmocodynamics.

Further studies have investigated the use of erythrocyte membranes themselves in drug delivery by coating the synthetic nanocarriers. RBCs’ blood groups serve as protection from the body’s immune system, making unnecessary the addition of molecules such as polyethylene glycol (PEG) to increase the drug carrier lifetime in circulation [17]. Even with the addition of PEG, synthetic liposomes only exhibit a half-life in blood of 3–6 h, significantly less than the RBC half-life of 10–15 days. Thus, if membrane extraction is performed properly, all of the proteins providing RBC immunity should be preserved and therefore nanocarriers coated with RBC membranes should maintain similar levels of immune protection. Biodegradable polymeric nanoparticles coated with erythrocyte membranes showed an increase in half-life to 39.6 h compared to the 15.8 h achieved with the PEGylated formulations [21].

The fact that nanocarriers coated with erythrocyte membranes show improved biocompatibility suggests that the erythrocyte membrane itself can be used as a liposome. The erythrocyte liposomes can be further optimized for molecule encapsulation by incorporating small amounts of synthetic lipids to produce hybrid erythrocyte membranes. Methods have been developed to allow for efficient incorporation of synthetic lipids by drying and incubating the hybrid membranes to allow membrane fusion from both synthetic and RBC domains to produce homogeneous membranes with no indication of phase separation [22,23,24]. Two examples are presented below to highlight particular erythrocyte liposomes for application in immunization and infectious diseases.

**Figure 2:**
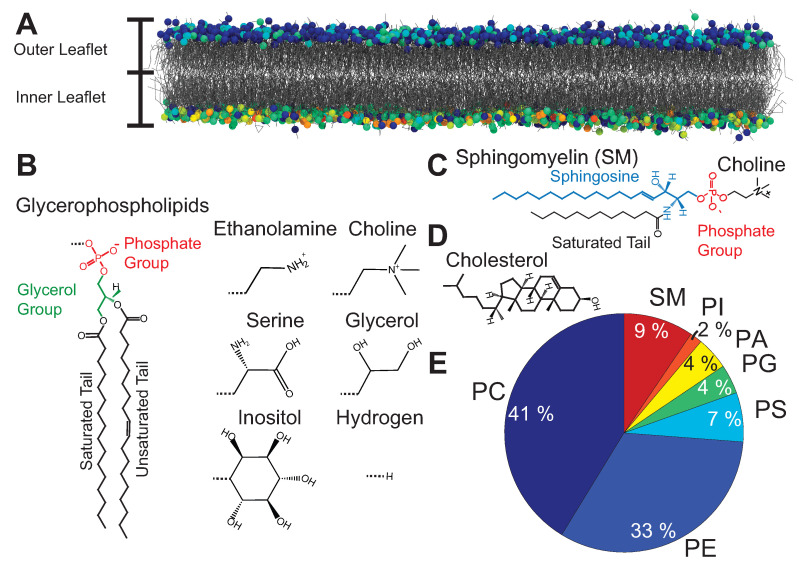
(**A**) A lipid membrane is formed by two layers (leaflets) of lipids molecules. Most membranes are built asymmetrically. (**B**) Glycerophospholipids and sphingomyelin consist of a hydrophilic head group and two hydrophobic tails. (**B**–**E**) Chemical structures of glycerophospholipids, sphingomyelin, and cholesterol. Common head groups are: choline, ethanolamine, serine, glycerol, inositol, and hydrogen. (**E**) Lipid distribution of the RBC cytoplasmic membrane. Figure adapted from [28].

The RBCs’ outer shell consists of a spectrin network tethered to a cytoplasmic membrane [25] (RBC_*cm*_RBC_*cm*_). The cytoskeleton forms a triangular filament network parallel to the RBC_*cm*_. The distance between tethers is ∼80 nm [25]. The RBC_*cm*_ is typically described by the fluid mosaic model [26], which describes this structure as a two-dimensional fluid-like lipid bilayer with embedded proteins. More than 50 of these membrane proteins have been characterized for the RBC_*cm*_ [27]. The lipid bilayer is a ∼5 nm [24] thick membrane formed by two layers (leaflets) of lipid molecules (Figure 2A). Membrane lipids are amphiphilic, i.e., they consist of a hydrophilic and a hydrophobic part. The molecules orient themselves such that the hydrophobic parts of both leaflets face towards each other while the hydrophilic parts are exposed to the aqueous environment. There is a large number of different types of lipids in this mammalian membrane mostly represented by glycerophospholipids (PL), sphingomyelin (SM), and cholesterol.

PLs are built around a glycerol moiety. Two of the carbon atoms are esterified to two fatty acids chains (tails) with the third carbon atom bound to a polar head group (see Figure 2B) [29]. Common head groups include choline, ethanolamine, serine, glycerol, inositol, and hydrogen. The fatty acid tails can vary in length, i.e., the number of carbon atoms per tail, and in the degree of saturation, i.e., the number of double bonds between the carbon atoms in the tail [29]. SM is built around sphingosine with an attached fatty acid chain and a phosphocholine head group (Figure 2C). PL and SM, cholesterol consists of a rigid structure formed by hydrocarbon rings (Figure 2D). Cholesterol is highly abundant in eukaryotic cell membranes, with typical molar ratios between 20 mol% and 50 mol% [30].

The lipid composition (lipidomics) of the RBC_*cm*_ has been determined by mass spectroscopy [31,32]. The abundance of PL and SM is shown in Figure 2E. Phosphatidylcholine (PC) and Phosphatidylethanolamine (PE) glycerophospholipids are the most abundant species in the membrane, followed by SM. Phosphatidylserine (PS), Phosphatidylglycerol (PG), Phosphatidic Acid (PA); Phosphatidylinositol (PI) lipids account for ∼20 % of the membrane. Importantly, these lipids are asymmetrically distributed between the two leaflets [27]. PC and SM lipids are predominantly found in the outer leaflet of the membrane while the majority of PE and PI lipids as well as all PS and PG lipids are located on the inner leaflet [27]. The cholesterol content of the RBC_*cm*_ has been reported to be ∼50 mol% [33,34]. The composition of this biological membrane is far more complex than synthetic membranes, which are typically composed of a couple of different lipid species, only, which is reflected in their complex structural and mechanical properties [28]. The advantages of RBC-based carriers are their high biocompatibility, low immunogenicity, and long systematic circulation. While synthetic liposomal carriers have lifetimes of some hours, RBC-based systems have shown to circulate for several weeks [35].

## 4. Implications for Immunology

Foreign particles introduced into the body’s circulation are typically rapidly degraded by the mononuclear phagocyte system (MPS) through phagocytosis. Nanoparticles in particular, will be tagged by MPS for uptake through opsinization [36]. As a result, nanoparticles are rapidly eliminated in the body with only a small fraction of the administered dose reaching the target cell. Efforts have been made to increase their longevity through the addition of polyethylene glycol (PEG) polymers on the nanoparticle’s exterior to produce a hydration layer that makes them no longer recognizable by the MPS [36]. Despite the potential of using PEG in these nanoparticle formulations, PEGylated liposomes are still limited in their life span.

The body’s immune system additionally consists of natural killer (NK) cells that function in eliminating target cells in response to particular ligands presented on their exterior [37]. Red blood cells, however, express the integrin-associated protein CD47 that serves as a marker-of-self to provide protection against the immune system. As a result, red blood cells can survive in circulation for up to 120 days. Moreover, scientists have been investigating the incorporation of CD47 as a replacement or addition to PEGylated synthetic nanoparticles with the hopes of increasing their circulation time. Studies have shown that nanoparticles conjugated to self-peptides designed from CD47 show a delay in macrophage-mediated clearance [38]. Incorporation of CD47 into synthetic nanoparticles requires additional optimization to ensure that the right levels of CD47 are incorporated to achieve the highest level of immune protection possible. In this sense, erythrocyte-based carriers present an advantage, as they already express natural levels of CD47 and thus maximum immune protection is established.

## 5. Erythrocte-Based Virus-like Particles

The outbreak of the coronavirus disease 19 (COVID-19) has challenged and still challenges the world in an unprecedented manner. It has led to over 640 million infections and more than 6.6 million deaths globally [39] (as of November 2022). The adverse effects of this global crisis, which has permeated all aspects of day-to-day living, including personal life, economy, and health care systems, substantiates an urgent need for novel diagnostics, therapeutics, and vaccines.

The severe acute respiratory syndrome-coronavirus-2 (SARS-CoV-2) is mainly transmitted via respiratory droplets [40,41]. In the lung, both SARS-CoV-2, as well as its precursor SARS-CoV, primarily infect the ciliated bronchial epithelial cells and type 2 pneumocytes [42,43,44] through the angiotensin converting enzyme 2 (ACE-2). This triggers a cascade of reactions leading to the fusion of the virus with the host cell and its reproduction, ultimately causing COVID-19. Of the three protein components on the viral envelope, the spike (S-)protein binds to the human ACE-2 receptor with a high affinity [45,46,47,48] and catalyses the viral and host membrane fusion to initiate the infection [48,49]. It is a densely glycosylated transmembrane protein that forms the characteristic surface spikes of the corona virus [48]. The protein also induces neutralizing antibody and T-cell responses, and is, therefore, an important target for vaccine development [50]. The structure and conformations of the SARS-CoV-2 S-protein have been elucidated, however, this is still a highly active field of research [45,47,49]. The basic structure consists of an ectodomain trimer that includes the receptor binding domain (RBD), a trans-membrane domain (TMD), and a cytoplasmic domain (CPD).

Several SARS-CoV-2 vaccines have been developed [51,52]. Gene-based vaccines deliver gene sequences that encode protein antigens that are produced by host cells. These include recombinant vaccine vectors (including AstraZeneca, Johnson & Johnson), or nucleic acid vaccines (including Pfizer/BioNTech, Moderna) [53]. The mRNA vaccines have shown a high potency [54] and typically require carriers, such as nanoparticles, as mRNA is quickly degraded by cellular processes.

In an alternative approach, the S-protein can be administered by the in vitro functionalisation of RBCs through directly anchoring the SARS-CoV-2 S-protein into the RBC_*cm*_. Nanocarriers adsorbed on RBCs have been shown to improve delivery for a wide range of carriers and viral vectors [55,56] and their biocompatibility may be advantageous over synthetic carriers [57,58]. However, their potential for therapeutic applications, such as drug delivery [59,60] and immunological functions [61,62,63,64] has been started to be exploited only recently. RBCs have been reported previously to catch immune complexes and bacteria and present them to Kupffer cells in the liver and Antigen-Presenting Cells (APCs) in the spleen [65,66]. Through this mechanism, virus-like particles (VLPs) prepared using RBCs (Erythro-VLPs) can potentially lead to antibody production, higher central memory T cell levels, and lower regulatory T cell response [67] when delivered to the spleen.

**Figure 3:**
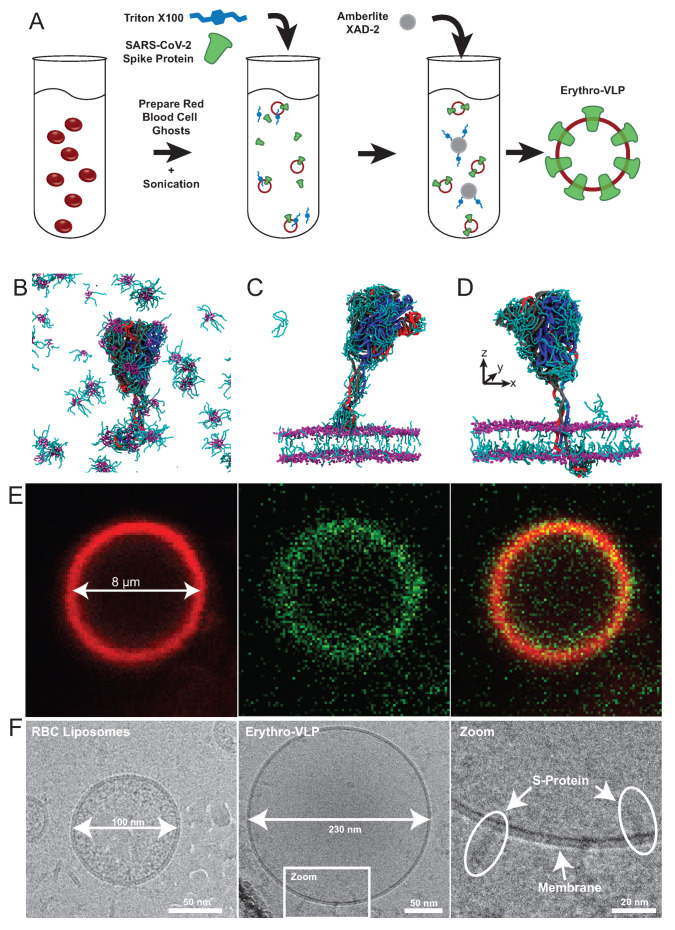
(**A**) Preparation protocol for Erythro-VLPs: Erythrocyte liposomes were prepared from human RBCs. Erythrocyte liposomes were incubated with S-protein in a surfactant (Triton-X 100) solution to facilitate protein entry into the membranes. Triton-X 100 was then removed using Amberlite XAD-2 resin beads and subsequent size-exclusion chromatography. (**B**) MD simulation show that the surfactant stabilizes the protein and protects the hydrophobic transmembrane domain. (**C**,**D**) show details of the insertion process. (**E**) Fluorescent microscope images of a giant Erythro-VLP with membrane stained in red, and S-proteins stained in green. (**F**) shows high-resolution cryo-TEM images of the erythrocyte liposomes before protein insertion, and with S-proteins embedded. Figure adapted from [35].

Erythro-VLPs were produced as sketched in Figure 3A. Erythrocyte liposomes were prepared [24] and incubated with a 3 μM S-protein solution containing 25 mM Triton-X 100 to reconstitute the S-protein in the membrane. The surfactant was afterwards removed by Amberlite XAD-2 resin beads [68], and subsequent size-exclusion chromatography (SEC). These techniques have become standard for Triton-X 100 removal [69]. The transport of the the CPD across the hydrophobic membrane core is essential in this step to anchor the S-protein in the membrane of the erythrocyte liposomes. The role of the surfactant is to stabilize the S-protein’s structure in the aqueous environment before insertion and to facilitate reconstitution of the S-proteins in the erythrocyte membranes, as shown in Figure 3B–D. From coarse grained Molecular Dynamics (MD) simulations, the surfactant binds to the protein in solution, particularly to the TMD and CPD and stabilizes the protein’s secondary structure by shielding the hydrophobic TMD. When the S-protein is close to the membrane (in Figure 3C), the CPD is the first point of contact. A high surfactant density is observed around the CPD, which facilitates insertion and passage through the membrane by lowering the hydrophobic mismatch between CPD and hydrophobic membrane core. Once the protein is fully anchored (Figure 3D), surfactant density around the TMD is significantly reduced and remains concentrated around the CPD and the surrounding inner leaflet. An embedding efficiency of 40% was determined, with an average protein density of ∼300 proteins/μm^2^ [35] and an average liposome diameter of 222 nm (polydispersity: 0.32). Successful conjugation of the S-protein with the erythrosome liposomes is shown using fluorescent microscopy and cryo-transmission electron microscopy (TEM). Figure 3E shows a giant Erythro-VLP, where the membrane was stained with Texas red 1,2-dihexadecanoyl-sn-glycero-3-phosphoethanolamine (TR-DHPE, red) and the S-proteins were stained using Alexa Fluor 488 maleimide (AF488, green). Membrane and proteins are then observed in the red and green channel, respectively, and the orange color in the combined channel is the result of the superposition of the red and green dyes and the images indicate a uniform distribution of the S-proteins in the erythrocyte membranes, while the RBC liposomes have a size of 100 nm in the cryo-TEM images in Figure 3F, the the Erythro-VLPs have sizes of ∼230 nm. The high-resolution images show S-proteins anchored with their TMD in the erythrocyte cytoplasmic membrane. The efficacy of the Erythro-VLPs was shown in a mouse study over a period of 33 days which showed seroconversion in vivo. While the mice received Erythro-VLP with the full-length S-protein, antibodies to the S-protein’s RBD sub-domain were measured, which is relevant for viral entry [70,71]. This implies that the conformation of the S-protein in the Erythro-VLPs is not changed in such a way that the RBD domain is ‘hidden’ or modified, which is often challenging when injecting soluble proteins [53]. An interesting point is that IgG production was triggered without an adjuvant (such as aluminium hydroxide [72,73]), which points to some sort of a depot effect, likely related to the circulation of the Erythro-VLPs in the blood stream before they are processed in the spleen.

## 6. Erythrocyte Liposomes for the Targeted Delivery of Antibiotics

The emergence of the antibiotic resistance crisis is the product of antibiotic overuse in the medical and industrial settings [74]. With increased exposure to antibiotics, bacteria are faced with selection pressures, allowing for the development and rapid spread of resistant mutations [75]. Without immediate global intervention and management, bacteria may evolve to develop multi-drug resistance (MDR), where infections once curable by common antibiotics become difficult to treat [76]. Enterobacteriaceae bacteria, such as *E. coli* and *Klesbiella* spp., are responsible for many serious infections: pneumonia, gastroenteric, and blood-stream infections [76]. Enterobacteriaceae are the major players in MDR and are thus a target species in the development of novel antimicrobial agents. In this context, polymyxin antibiotics are able to treat Gram-negative bacterial infections with high efficacy [77]. Despite being highly potent, polymyxin B (PmB) is considered a last resort treatment due to its toxic side effects such as nephrotoxicity, neurotoxicity, and neuromuscular blockade [78,79,80]. Studies have thus been focused on optimizing the PmB dosage for each patient to minimize toxicity with high efficacy [10,77,81].

Nanoparticles have been used deliver PmB to the infection site with the goal to reduce its random distribution throughout the body. Proteins or ligands can be conjugated to the nanoparticle surface to achieve targeted delivery, where an entry mechanism to the target bacterial cell is required for antibiotic delivery [3]. Metallic nanoparticles themselves show antimicrobial properties through the release of bactericidal free metal ions, the production of free radicals, and interactions with the bacterial DNA [82]. The antimicrobial activity of silver and gold nanoparticles could be further improved through functionalisation with ampicillin to induce bactericidal activity against bacteria with *β*-lactam resistance [83]. Gold nanoparticles functionalised with carbapenems showed antimicrobial activity in vitro against MDR bacterial strains [84].

On the other hand, liposomes have been shown to deliver antibiotics through a fusion mechanism with the bacterial membrane, allowing high antibiotic concentrations to be delivered at low dosages. Electrostatic interactions likely play an important role in this process as biofilm formation was reduced when positively charged clarithromycin was encapsulated in negatively charged liposomes. However, positive liposome formulations required smaller clarithromycin dosages, reducing toxicity [85]. Further optimization of the membrane may be required to improve retention of the desired molecules. For instance, liposomes composed of dipalmitoylphosphatidylcholine/cholesterol and palmitoyloleoylphosphatidylcholine/cholesterol showed loading efficiencies on the order of a few percent [86,87]. Loading could be further increased to ∼50% with formulations with varying ratios of phosphatidylcholine, sphingomylein, and cholesterol [88].

**Figure 4:**
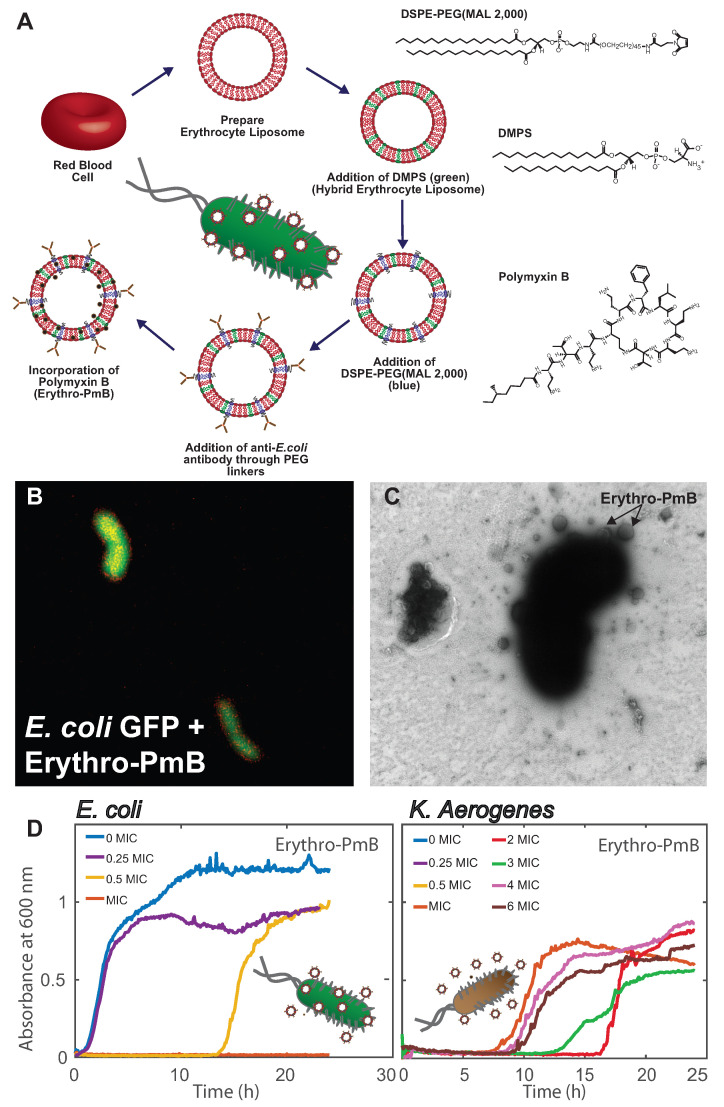
(**A**) Preparation schematic for Erythro-PmBs: Erythrocyte liposomes were prepared from red blood cells. Negatively charged lipids were added to enhance PmB retention. Anti-*Escherichia coli* (*E. coli*) antibodies are conjugated to malemeide residues on PEG linkers. (**B**) Fluorescent microscopy image of Erythro-PmBs stained with TR-DHPE (red) targeting *E. coli* expressing green fluorescent protein (GFP, green). (**C**) Erythro-PmBs form attachments with *E. coli* in transmission electron microscopy. (**D**) *E. coli*, (**left**) and *Klebsiella aerogenes* (*K. aerogenes*, (**right**)) are treated with Erythro-PmBs delivering varying concentrations of the minimum inhibitory concentration (MIC) for free PmB. Bacterial growth curves show Erythro-PmBs prevent *E. coli* growth with high efficacy; however, are not active against *K. aerogenes*. Figure adapted from [89].

More recently, hybrid erythrocyte liposomes were prepared by doping the RBC_*cm*_ with small amounts of 1,2-dimyristoyl-sn-glycero-3-phospho-L-serine (DMPS) as shown in Figure 4A, to enhance retention of the cationic PmB [89]. Incorporation of negative charges achieved an increased loading efficiency of ∼90%, suggesting that retention is dominated by electrostatic attractions between the PmB molecules and the membrane lipids. While PmB is known to interact with bacterial membranes through insertion [90,91], the presence of cholesterol in the erythrocyte membrane was shown to prevent membrane collapse by stabilizing the bilayer structure.

These Erythro-PmBs were made specific to *E. coli* bacteria through conjugation of anti-*E. coli* antibodies to 1,2-distearoyl-sn-glycero-3-phosphoethanolamine-N-[maleimide (polyethylene glycol)-2,000] (PEG-MAL(2,000)) lipids incorporated in the erythrocyte membrane, as shown in Figure 4A. This step requires the reduction of interchain disulfide bonds in the antibody to form reactive cysteine residues that form bonds with the malemeide groups on the PEG terminal. Erythro-PmBs stained with TR-DHPE (red) attached to *E. coli*—Green Fluorescent Protein (GFP) in Figure 4B, where the yellow color is indicative of Erythro-PmB colocalization. Erythro-PmBs were further incubated with *E. coli* and imaged with TEM in Figure 4C, where Erythro-PmBs were found to concentrate around the *E. coli* and form attachments to the bacterial surface.

These Erythro-PmBs could deliver PmB to non-resistant *E. coli* with similar efficacy to that of free PmB, as indicated by no change in the minimum inhibitory concentration (MIC) in Figure 4D. Antibody conjugation, however, established specificity as PmB was not effectively delivered to the bacterial strain *Klebsiella aerogenes* that lacked the corresponding antigens. *K. aerogenes* still exhibited growth after higher orders of the MIC, indicating that the bacteria must display the proper antigens in order for an interaction between the Erythro-PmBs and bacterial membrane to occur thereby delivering PmB.

## 7. Current Limitations and Future Perspective

There is potential for the erythrocyte-based platform to be transferred to other types of therapeutic molecules; however, some considerations must be addressed. Firstly, the interaction of the cargo with the erythrocyte membrane will determine the encapsulation procedure required. If the cargo is membrane-active, such as polymyxin B, it may be incorporated into or on the surface of the outer-leaflet of the erythrocyte membrane. Conversely, hydrophilic molecules will need to be encapsulated within the erythrocyte liposome’s aqueous core. Secondly, the properties of the cargo molecule will reflect the type of synthetic lipids that must be incorporated in the hybrid membrane in order to achieve optimal loading without compromising the membrane structure. Such properties may include molecular charge, size, and stereochemistry.

There are additionally some considerations that must be taken prior to establishing the erythrocyte-based platform as a therapy option in the clinical setting. Prior to becoming a commercial product, future work is required to determine large-scale manufacturing of the erythrocyte liposomes. For instance, this may involve the use of microfluidic devices. While this erythrocyte-based platform presents an advantage in terms of biocompatibility, further research is required to determine the importance of the blood types prior to administration in human patients. As of right now, it is unclear whether the erythrocyte liposomes must be derived from blood-types compatible to that of the patient, or whether blood types become negligible. This will become particularly important when doing large-scale production. If blood type is indeed relevant, then a more personalized medicinal approach may be taken where the donor blood comes from the patient themselves.

**Figure 5:**
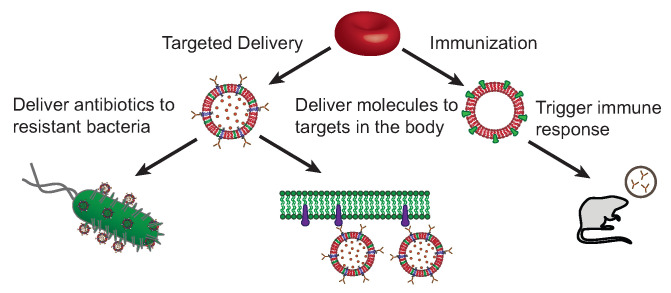
Erythrocyte-based drug delivery platform. (**Left**) Erythrocyte liposomes are produced from red blood cells and their molecular structure and properties tuned by incorporation of synthetic lipids. Specificity is achieved through conjugation of corresponding antibodies to deliver molecules to various targets. For instance, antibiotic resistant bacteria, tumours, or transport proteins in the blood–brain barrier. (**Right**) Virus-like particles can be generated through anchoring of viral antigens in the liposomal membranes to trigger an immune response.

Both systems, the Erythro-VLPs and the Erythro-PmBs (Figure 5) are examples of a novel, blood-based platform of therapeutics. This platform should easily be expanded to include for instance applications in the treatment of cancer by conjugating the erythrocyte liposomes with antibodies that target tumours and cancers cells, and delivering anti-tumour drugs. By conjugating with antibodies targeting receptors or transporters in the blood–brain-barrier (BBB), the platform can also potentially help to deliver molecules, such as anti-dementia drugs, across the BBB. The target proteins may attach to receptors or transporters in the BBB and are capable to deliver the load across the BBB and into the brain.

While first results for the Erythro-VLPs demonstrate the potential of this pathway and the erythrocyte platform, future work is needed to establish its potential therapeutic use. This includes, for instance, in vivo toxicity evaluations and pathological analysis including vasculitis, and options for intramuscular administration. Seroconversion was accomplished by using a virus-like-particle emerging from red blood cells on the one hand, which needs to be carefully tested in vivo. The platform also uses a relatively unexplored pathway to produce antibodies and potential vaccination that involves a different immune response as compared to most current vaccines. As such, the exact retention time and location of the VLPs in organisms, and their immunological pathway needs to be explored in detail to fully evaluate their potential and identify potential drawbacks and risks. Potential advantages of this pathway certainly lay in the enhanced biocompatibility and promise of reduced side effects and potentially high social acceptance because it does not involve genetics. The platform is also versatile and can be adapted to new strains or viruses quickly by embedded the corresponding antigens.

The use of nanoparticles as a delivery system for antibiotics has been explored as a solution because they have the potential to penetrate through thick mucus layers and biofilms produced by bacteria [92]. Additionally, nanoparticles may allow for the localized delivery of higher antibiotic concentrations to the infection site, which would reduce the patient’s exposure to the antibiotic throughout treatment. Liposomes composed of phosphatidylcholine (PC) lipids encapsulating the antibiotic ciproflacin exhibited a sustained release of ciproflacin to model lung cells [93]. Achieving sustained release, allows for the antibiotic to be delivered to the target site for a prolonged period of time rather than administering multiple doses and therefore, has potential to reduce negative side effects. PC liposomes carrying amikacin showed increased penetration into *Pseudomonas* biofilms with higher concentrations delivered [94]. Despite their potential, liposomes are sensitive to the method of aerosolisation and are easily susceptible to membrane degradation and aggregation if not chosen properly. As a result, a large fraction of the encapsulated antibiotic is released from the liposome within the aerosol droplet, where the free antibiotic is now taken up by the antibiotic. Additionally, liposomes themselves are limited in their physical instability and rapid clearance by the immune system. Using RBCs for aerosol delivery may resolve the limitations associated with synthetic liposomes. Their high biocompatibility would result in protection from the body’s immune system, allowing them to remain in circulation for longer, further reducing the need for multiple dose administrations. The RBCcm composition is comprised of ∼ 50% cholesterol which may provide additional physical support during aerosoliza tion, reducing the likelihood for membrane damage.

## Data Availability

No new data were created or analyzed in this study. Data sharing is not applicable to this article.

## Abbreviations

RES	Reticuloendothelial System
PEG	Polyethylene Glycol
RBC	Red Blood Cell
TM	Thrombomodulin
*E. coli*	*Escherichia coli*
ASNase	L-asparaginase II
RBC_*cm*_	Cytoplasmic RBC Membrane
PL	Glycerophospholipid
SM	Sphingomyelin
PC	Phosphatidylcholine
PE	Phosphatidylethanolamine
PS	Phosphatidylserine
PG	Phosphatidylglycerol
PA	Phosphatidic Acid
PI	Phosphatidylinositol
MPS	Mononuclear Phagocyte System
SARS-CoV-2	Severe Acute Respiratory Syndrome—Coronavirus-2
APC	Antigen Presenting Cell
VLD	Virus Like Particle
SEC	Size Exclusion Chromatography
ACE-2	Angiotensin Converting Enzyme 2
RBD	Receptor Binding Domain
TMD	Transmembrane Binding Domain
MD	Molecular Dynamics
TEM	Transmission Electron Microscopy
TR-DHPE	Texas Red 1,2-Dihexadecanoyl-sn-glycero-3-phosphoethanolamine
AF488	Alexa Fluor 488
DMPS	Dimyristoyl-sn-glycero-3-phospho-L-serine
PmB	Polymyxin B
GFP	Green Fluorescent Protein
MIC	Minimum Inhibitory Concentration
*K. aerogenes*	*Klebsiella aerogenes*
